# Supplementary material for: Opal Actigraphy (Activity and Sleep) Measures Compared to ActiGraph: A Validation Study
Source: Sensors (Basel). 2023 Feb 18;23(4):2296. doi: 10.3390/s23042296 (PMC10003936; doi:10.3390/s23042296)
Supplement: Supplementary file 1 [file sensors-23-02296-s001.zip › sensors-2118699-supplementary.pdf]

Table S1. Activity and sleep measures average across 7 days of all participants for the four sensors.

Metric	Opal versus ActiGraph GT9X (non-dominant arm)		Actigraph GT3X versus ActiGraph GT9X (dominant arm)	
**Activity measures (Freedson 98)**	**Opal**	**ActiGraphGT9X**	**ActiGraphGT3X**	**ActiGraphGT9X**
Total counts (#)	1,134,078.00 (347,558.80)	1,157,470.00 (350,752.00)	1,294,464.00 (431,727.20)	1,280,843.00 (418,138.40)
Light activity(min)	735.95 (81.05)	732.65 (81.29)	704.23 (90.17)	707.19 (91.50)
Moderate activity (min)	482.43 (62.15)	479.66 (62.50)	478.71 (77.15)	477.04 (75.70)
Vigorous activity (min)	211.71 (67.63)	217.07 (68.49)	243.23 (84.54)	242.58 (83.78)
Very vigorous activity (min)	7.41 (7.58)	8.02 (8.12)	11.27 (10.92)	10.70 (9.74)
Sedentary activity (min)	2.50 (6.78)	2.61 (6.92)	2.56 (6.91)	2.49 (6.91)
**Activity measures (Freedson VM3)**	**Opal**	**ActiGraphGT9X**	**ActiGraphGT3X**	**ActiGraphGT9X**
Total counts (#)	2,168,544.00 (556,343.80)	2,213,815.00 (568,297.20)	2,412,999.00 (645,513.40)	2,386,703.00 (633,010.50)
Light activity(min)	1123.21 (81.15)	1117.31 (82.48)	1093.30 (94.40)	1095.46 (93.76)
Moderate activity (min)	243.95 (57.96)	247.38 (59.77)	243.49 (62.22)	244.79 (63.06)
Vigorous activity (min)	64.19 (37.74)	65.34 (38.64)	91.01 (47.09)	88.41 (45.97)
Very vigorous activity (min)	8.64 (10.65)	9.97 (11.80)	12.20 (12.58)	11.34 (11.88)
**Sleep measures**	**Opal**	**ActiGraphGT9X**	**ActiGraphGT3X**	**ActiGraphGT9X**
Total sleep time (min)	354.02 (111.72)	346.71 (109.23)	407.93 (88.33)	349.42 (111.80)
Wake after sleep onset (min)	49.33 (18.85)	37.18 (14.47)	30.47 (12.16)	34.06 (12.52)
Awakenings (#)	9.97 (4.78)	15.16 (7.06)	12.89 (5.31)	14.23 (5.49)
Avg. awakening Length (min)	2.73 (0.97)	2.64 (0.67)	2.47 (0.58)	2.56 (0.71)
Sleep efficiency (%)	87.06 (5.71)	89.74 (4.08)	92.70 (3.38)	90.22 (3.83)
Total counts (#)	21,004.78 (7355.67)	18,683.27 (6964.99)	16,476.95 (5190.29)	17,077.20 (5608.85)

Table S2. Sensor characteristics for Opal, GT9X and GT3X.

Specification	Opals	GT9X	GT3X
Dimensions	50 × 40 × 14 mm	4.18 × 3.98 × 1.13 cm (in holster)	4.6 × 3.3 × 1.5 cm
Weight	25 g	14 g	19 g
Sample rate	30 Hz	30-100 Hz	30-100 Hz
Accelerometer dynamic range	16 G	+/- 8G	+/- 8G
Data storage	8 GB	4GB	4GB
